# Analysis of the kidney failure risk equation implementation in routine clinical practice and health inequalities in chronic kidney disease care: a retrospective cohort study

**DOI:** 10.1186/s12882-025-04043-0

**Published:** 2025-03-04

**Authors:** Heather Walker, Shabana Khan, Sandosh Padmanabhan, Jill P. Pell, Jim Lewsey, Daniel Mackay, Ruth Dundas, Jocelyn M. Friday, Tran Q. B. Tran, Denise Brown, Frederick Ho, Claire E. Hastie, Michael Fleming, Claudia Geue, Alan Stevenson, Clea Du Toit, Bhautesh Dinesh Jani, Katie Gallacher, Patrick B. Mark, Michael K. Sullivan

**Affiliations:** 1 School of Cardiovascular and Metabolic Health, University of Glasgow, Glasgow, Scotland; 2 Robertson Centre for Biostatistics, University of Glasgow, Glasgow, Scotland; 3 Public Health, School of Health and Wellbeing, University of Glasgow, Glasgow, Scotland; 4 Health Economics & Health Technology Assessment, School of Health and Wellbeing, University of Glasgow, Glasgow, Scotland; 5 MRC/CSO Social & Public Health Sciences Unit, University of Glasgow, Glasgow, Scotland; 6 Digital Health Validation Lab, Living Lab, University of Glasgow, Glasgow, Scotland; 7 General Practice and Primary Care, School of Health and Wellbeing, University of Glasgow, Glasgow, Scotland; 8 Renal and Transplant Unit, Queen Elizabeth University Hospital, Glasgow, Scotland

**Keywords:** Chronic kidney disease, Albuminuria, Kidney failure risk equation, Hospital referrals, Health inequality

## Abstract

**Background:**

NICE guidelines recommend GPs use the kidney failure risk equation (KFRE) to identify people with chronic kidney disease (CKD) at higher risk of kidney failure. Albuminuria results are required to calculate KFRE.

**Aim:**

Analyse the implementation of KFRE into clinical practice and investigate if albuminuria testing varied amongst patients with CKD, particularly for underserved groups.

**Design and setting:**

Retrospective cohort study of 23,063 adults in Glasgow from 2013 to 2022.

**Method:**

We evaluated albuminuria testing rates and the predictive performance of KFRE in estimating 5-year kidney failure risk amongst people with CKD. Logistic regression models quantified associations between demographic/clinical variables and albuminuria testing. Amongst people who developed kidney failure, we retrospectively assessed the impact of KFRE on the timing of meeting criteria for referral to renal services.

**Results:**

Albuminuria testing was performed in 44.5% of 10,874 adults with CKD. Females (adjusted odds ratio (aOR) 0.86: 95% CI 0.79–0.93) and those with hypertension (aOR 0.69: 95% CI 0.63–0.77) were less likely to have albuminuria testing. Those aged 40–50 years (aOR 1.83: 95% CI 1.15–2.91), with diabetes (aOR 2.35: 95% CI 2.14–2.58) and living in the least socioeconomically deprived areas (aOR 1.11: 95% CI 1.00-1.23) were more likely to have albuminuria testing. Of 1,352 individuals with incident kidney failure, incorporating KFRE into referral guidelines helped identify high-risk patients early.

**Conclusion:**

KFRE could be calculated for less than half of people due to lack of albuminuria testing. Focus should be given to improving albuminuria testing and inequities identified to allow wider implementation of KFRE.

**Supplementary Information:**

The online version contains supplementary material available at 10.1186/s12882-025-04043-0.

## Introduction

Chronic kidney disease (CKD) is common, affecting approximately 10% of the population globally [1]. Although most people with CKD have mild disease with a slight reduction in estimated glomerular filtration rate (eGFR), a small proportion of people develop kidney failure requiring kidney replacement therapy (KRT: long-term dialysis or kidney transplantation). Identifying individuals with CKD at risk of progressing to kidney failure can be challenging and in the past, referrals from primary to secondary care primarily relied on eGFR thresholds (e.g. eGFR < 30 mL/min/1.73m^2^), resulting in some low-risk patients being referred [2]. Risk prediction models– such as the kidney failure risk equation (KFRE)– aim to estimate the risk of kidney failure for an individual using clinical and demographic information [3]. One of the main aims of prognostic models, like the KFRE, is to aid treatment decisions and to more accurately identify adults with CKD at elevated risk of kidney failure [3].

Clinical guidelines in the UK from The National Institute for Health and Care Excellence (NICE) incorporated the KFRE into CKD guidelines in 2021 [4], suggesting that adults with a five-year risk of kidney failure greater than 5% be referred for specialist assessment. The KFRE is a simple tool, using age, sex, eGFR and urine albumin-creatinine ratio (uACR). However, uACR testing amongst people with CKD has historically been low in the UK [5] and globally [6, 7], which challenges the implementation of KFRE in clinical practice.

In this study, we hypothesised that low uACR testing rates would be observed amongst patients with CKD, particularly for underserved groups such as women or those from more socioeconomically deprived areas [8]. We sought to ascertain whether updated NICE guidelines from 2021, if used, would identify individuals at elevated risk of kidney failure earlier than older guidance which relied primarily on eGFR. We used data from a large population with linkage to renal health records, which allowed us to reliably identify CKD and kidney failure. We aimed to discover (1) what demographic variables were associated with availability of uACR results, (2) if referral criteria which included KFRE identified patients earlier than criteria based mainly on eGFR and (3) the predictive performance of KFRE for individuals who had uACR results available.

## Methods

### Study design and setting

A retrospective cohort study was performed using data from the West of Scotland Safe Haven. This holds data on approximately 1.4 million people living within the Greater Glasgow & Clyde area, UK [9]. Haematology and biochemistry results were obtained from the Scottish Care Information (SCI) Store [10], renal data from the Strathclyde Electronic Renal Patient Register (SERPR, Vitalpulse, UK) which records data on all individuals referred to the renal service and all renal replacement therapy and transplantation activity, and mortality data from the National Records of Scotland. These data were also linked to hospital admissions (Scottish Morbidity Record 01 (SMR01)) and the SCI diabetes register [11]. We studied adults aged 18 years and over. The study period started on January 1st, 2013, as before this date we could not be certain that laboratory testing of serum creatinine used standardised instruments. The follow-up period extended until the 31st of December 2022. The study is reported in line with Strengthening the Reporting of Observational Studies in Epidemiology (STROBE) guidelines [12] and Transparent reporting of a multivariable prediction model for individual prognosis or diagnosis (TRIPOD) guidelines [13].

### Variables and definitions

Age to the nearest year, was defined at the date of CKD identification, based on the latest sustained eGFR measurement < 60mL/min/1.73m^2^. Age was categorised (18–40, 40–50, 50–60, 60–70, 70–80 and > 80 years) to aid interpretation of results. Area-based socioeconomic deprivation was measured using the Scottish Index of Multiple Deprivation (SIMD) [14], categorised into tertiles (SIMD deciles 1–3, 4–7 and 8–10). The latest SIMD status (SIMD 2012) recorded before the start of the study period was used. Hypertension and diabetes diagnoses were ascertained from SMR01 and SCI diabetes records. Availability of albuminuria testing was identified by the presence of a uACR result six months before or after meeting CKD diagnostic criteria.

We ascertained which people met the criteria for referral to renal services, using two NICE guidelines. The referral criteria in the 2014 NICE guideline were [15]:

- eGFR < 30mL/min/1.73m^2^.
- uACR > 70 mg/mmol.
- A sustained decrease in eGFR of 25% or more and a change in eGFR category, or a sustained decrease in eGFR of 15mL/min/1.73m^2^ or more within 12 months.

The referral criteria in the 2021 NICE guideline were [4]:

- Five-year risk of requiring KRT exceeding 5% (estimated using KFRE using age, sex, eGFR, uACR [16])
- uACR > 70 mg/mmol.
- A sustained decrease in eGFR of 25% or more and a change in eGFR category, or a sustained decrease in eGFR of 15mL/min/1.73m^2^ or more within 12 months.

Both guidelines also included uACR > 30 mg/mmol combined with haematuria, but it was not possible to include this criterion as we did not have information on the presence or absence of haematuria.

Referral criteria were classed as being met if at least one criterion within the referral criteria was met. Individuals were then categorised as meeting the 2014 or 2021 guideline, both or neither. For people who met both guideline referral criteria, as described above, we identified which guideline referral criteria was met first or if they were met at the same time (where time zero is the date of meeting the criterion). We quantified the rates of incident kidney failure and all-cause mortality for this population. Incident kidney failure was defined as commencing dialysis for 90 days or more, receipt of a kidney transplant or eGFR decline to < 15mL/min/1.73m^2^ sustained for more than 90 days; whichever occurred first.

### Statistical methods

Baseline characteristics were described using means with standard deviations (SD) for continuous data, medians with interquartile ranges (IQR) for non-normally distributed data and counts with percentages for categorical vairables. Analyses were performed using R (version 4.3.1).

### Analysis 1– Application of KFRE to people with CKD

People with CKD G3-5 with at least five years of follow-up were included, i.e., they had two eGFR values < 60mL/min/1.73m^2^ more than 90 days apart with no values > 60mL/min/1.73m^2^. The eGFR was calculated from creatinine using the CKD-EPI 2009 equation without incorporating the race coefficient [17], as recommended during the study period. People on long-term dialysis or previous kidney transplantation were excluded.

We performed univariable and multivariable logistic regression models, including age, sex, SIMD, diabetes and hypertension diagnoses, to quantify associations between predictor variables and the availability of uACR results. Incidence of kidney failure and all-cause mortality were expressed per 1000 patient-years. Individuals were censored at time of death, reaching kidney failure or last recorded activity in health records, whichever occurred earliest.

### Analysis 2– Value of KFRE compared to eGFR threshold < 30mL/min/1.73 m^2^ to guide referral for people who develop kidney failure

People with incident kidney failure and at least five years of preceding data were included. We identified which of these people had eGFR and uACR values available five years before the development of kidney failure.

The time from meeting referral criteria to the first kidney failure event, censored at the end of 1 year after meeting the guideline referral criteria, was explored. We performed Cox proportional hazards regression to assess associations with time delay from date of meeting referral criteria to onset of kidney failure. Numbers of individuals meeting referral criteria within 365 days before initiation of KRT (dialysis or transplant) was compared between individuals meeting 2014, 2021 or both 2014 and 2021 NICE guidelines referral criteria.

### Analysis 3– Predictive performance of KFRE

People in analysis 1 who had uACR values available within six months of meeting CKD diagnostic criteria were included. We calculated KFRE values for individuals and assessed the performance of the KFRE for predicting kidney failure events using (i) area under the receiver operating characteristic curve for discrimination and (ii) calibration curves for calibration [3].

## Results

### Analysis 1– Application of KFRE to people with CKD

Of 23,063 adults with CKD, 10,874 were included in the analysis (Additional File 1: Figure S1). There were 11,861 individuals excluded as their first eGFR measurement < 60mL/min/1.73m^2^ was outside of the study period. The median age at CKD diagnosis was 79 (IQR 71–85) years, and the median eGFR was 49.6 (IQR 39.4–45.1) mL/min/1.73m^2^ (Table 1).

**Table 1:** Baseline characteristics

		Analysis 1 (prevalent CKD)	Analysis 2 (incident chronic kidney failure during the study period)	Analysis 3 (prevalent CKD population [population 1] with uACR data)
Patients	*n*	10,874	1,352	4,838
**Sex**	Male	4,402 (40.5)	758 (56.1)	2,151 (44.5)
	Female	6,472 (59.5)	594 (43.9)	2,687 (55.5)
Age	Median (IQR)	79 (71–85)	64 (52–77)	77 (69–83)
eGFR mL/min/1.73 m^2^	Median (IQR)	49.6 (39.4–45.1)	11.5 (7.0–14.0)	48.6 (38.1–54.5)
Baseline eGFR (from median creatinine previous 365 days), n (%), mL/min/1.73 m^2^	≥60	1,713 (15.8)	29 (2.1)	646 (13.4)
	45–59	5,784 (53.2)	25 (1.8)	2,536 (52.4)
	30–44	1,900 (17.5)	32 (2.4)	898 (18.6)
	15–29	969 (8.9)	370 (27.4)	546 (11.3)
	<15	508 (4.7)	890 (65.8)	212 (4.4)
	Missing	N/A	6 (0.4)	N/A
**uACR** **Available within 6 months of CKD diagnosis**,** n(%), mg/mmol**	Median (IQR)	2.7 (0.6–14.7)	11.7 (1.0–78.0)	2.7 (0.6–14.7)
	<3	2,501 (23.0)	153 (11.3)	2,501 (51.7)
	3–30	1,473 (13.6)	137 (10.1)	1,473 (30.5)
	31–69	340 (3.13)	56 (4.1)	340 (7.0)
	≥70	524 (4.8)	129 (9.5)	524 (10.8)
	Missing	6,036 (55.5)	877 (64.9)	N/A
Hypertension*	n(%)	2,032 (18.7)	542 (40.1)	785 (16.2)
Diabetes*	*n*(%)	2,706 (24.9)	537 (39.7)	1,663 (34.4)
Socioeconomic deprivation Scottish index of multiple deprivation (SIMD) group	1–3 most deprived	4,822 (44.3)	652 (48.2)	2,196 (45.4)
	4–7	3,176 (29.2)	346 (25.6)	1,341 (27.7)
	8–10 least deprived	2,560 (23.5)	290 (21.4)	1,169 (24.2)
	Missing	316 (2.9)	64 (4.7)	132 (2.7)
Death	*n*(%)	7,108 (65.4)		2,894 (59.8)
Kidney failure/ESKD	*n*(%)	989 (9.1)	N/A 100%	594 (12.3)
	Dialysis	143 (1.3)	N/A	89 (1.8)
	Transplant	153 (1.4)	N/A	97 (2.0)
	eGFR < 15 mL/min/1.73m^2^	693 (6.4)	N/A	408 (8.4)
Baseline 5-year KFRE (%)	Median (IQR)	N/A	N/A	0.34 (0.09–1.9)
	<5		N/A	4,009 (82.9)
	5–9	N/A	N/A	202 (4.2)
	10–14	N/A	N/A	107 (2.2)
	15–20	N/A	N/A	70 (1.5)
	>20	N/A	N/A	450 (9.3)
	Missing	N/A	N/A	N/A

* Prior to first eGFR < 60 (population 1/3) prior to ESKD (population 2)

uACR was available in 44.5% of people. Univariable analysis showed that the people least likely to have uACR tested were females, those under the age of 40 years, those over the age of 80 years, those without diabetes, and those with hypertension (Table 2). Multivariable logistic regression showed characteristics independently associated with albuminuria testing were age 40–50 years (adjusted odds ratio (aOR) 1.83 (95% confidence interval (CI) 1.15–2.91), *p* = 0.01), a diagnosis of diabetes (aOR 2.35 (95% CI 2.14–2.58), *p* < 0.001) and living in the least socioeconomically deprived areas (aOR 1.11 (95% CI 1.00-1.23), *p* = 0.04). Characteristics independently associated with lack of uACR testing were female sex (aOR 0.86 (95% CI 0.79–0.93), *p* < 0.001) and diagnosis of hypertension (aOR 0.69 (95% CI 0.63–0.77), *p* < 0.001).

**Table 2:** Analysis 1 results. Numbers of participants with available uACR and logistic regression

		uACR available *n* (%)	Odds ratio (95% confidence interval)	
			Univariable	Multivariable*
**Sex**	Male	2,151 (48.9)	Ref.	Ref.
	Female	2,687 (41.5)	0.74 (0.69–0.80)	0.86-(0.79–0.93)
**Age group**	18–40	49 (44.1)	Ref.	Ref.
	40–50	139 (59.9)	1.89 (1.20–2.99)	1.83 (1.15–2.91)
	50–60	312 (53.4)	1.48 (0.99–2.23)	1.32 (0.87-2.00)
	60–70	770 (55.1)	1.55 (1.05–2.29)	1.41 (0.95–2.10)
	70–80	1,905 (50.4)	1.28 (0.88–1.88)	1.29 (0.88–1.90)
	> 80	1,663 (34.8)	0.68 (0.46–0.99)	0.75 (0.51–1.10)
**SIMD group** Missing values *N* = 316 (2.9%)	1–3 (most deprived)	2,196 (45.5)	Ref.	Ref.
	4–7	1,341 (42.2)	0.87 (0.80–0.96)	0.93 (0.84–1.02)
	8–10 (least deprived)	1,169 (45.7)	1.00 (0.91–1.11)	1.11 (1.00-1.23)
**Hypertension**	No	4,053 (45.8)	Ref.	Ref.
	Yes	785 (38.6)	0.74 (0.67–0.82)	0.69 (0.63–0.77)
**Diabetes**	No	3,175 (38.9)	Ref.	Ref.
	Yes	1,663 (61.5)	2.51 (2.29–2.74)	2.35 (2.14–2.58)

*Adjusted for sex, age group, SIMD group, hypertension and diabetes

Within five years of CKD diagnosis, 62.0% of people met one or more criteria for referral to specialist renal services. Of the 6,740 people who met a referral criterion, 5,318 (78.9%) met the 2014 and 2021 criteria on the same date. The 2021 criteria identified 413 people earlier than the 2014 criteria, which represented 6.1% of everyone who met a criterion at any time.

There were 989 people with incident kidney failure (long-term dialysis, transplantation or sustained eGFR < 15mL/min/1.73m^2^). Event rates per 1000 patient-years were 13.3 for eGFR < 15, 2.9 for kidney transplantation, 2.7 for long-term dialysis, and 136.0 for all-cause mortality.

### Analysis 2– Value of KFRE compared to eGFR threshold < 30mL/min/1.73 m^2^ to guide referral for people who develop kidney failure

Of 1,736 people with incident kidney failure, 1,352 were included as they had five years of preceding data (Additional File 1: Figure S2). The median age at the development of kidney failure was 64 years, and 56.1% were male (Table 1).

The first kidney failure event was ascertained by eGFR < 15mL/min/1.73m^2^ in 640 (47.3%) people, dialysis in 220 (16.3%), and transplantation in 492 (36.4%). Of these, 1,277 (94.5%) met one or both of the 2014/2021 NICE referral guidelines criteria (Table 3). The 2021 criteria identified 116 people earlier than the 2014 criteria, which represented 8.6% of the people who met criteria at any time. The 2014 criteria identified 239 (18.7%) people earlier than the 2021 criteria. Those who met 2021 referral criteria were more likely to meet the criteria ≥ 365 days before experiencing kidney failure compared to those who met the 2014 criteria (HR 0.11, 95%CI 0.02–0.83; *p* = 0.032). The mean time for meeting referral guideline criteria prior to kidney failure was 6.2 (SD 2.3) years when NICE 2021 criteria were met first compared to 3.9 (2.5) years when NICE 2014 criteria were met first.

**Table 3:** Individuals meeting NICE guideline referral criteria and timing in relation to experiencing kidney failure

NICE guideline	*n* (%) 1352	Meeting referral criteria > 365 days before kidney failure event *n*(%)	Time of meeting referral criteria before kidney failure event (years): mean (SD)
None	75 (5.6)	-	-
Met both on same date	893 (66.1)	826 (92.5)	5.0 (2.6)
NICE 2014 first	239 (17.7)	211 (88.3)	3.9 (2.5)
NICE 2021 first	116 (8.6)	113 (97.4)	6.2 (2.3)
NICE 2014 only	22 (1.6)	13 (59.1)	2.1 (2.1)
NICE 2021 only	7 (0.5)	1 (14.3)	0.2 (1.1)

### Analysis 3– Predictive performance of KFRE

Of 23,063 adults with CKD, 4,838 were included in the analysis (Additional File 1: Figure S3). The median five-year KFRE was 0.3% (Table 1).

The highest KFRE values were found amongst males, adults aged 40–50 years, people with diabetes, and people living in the most deprived areas (Table 4).

**Table 4:** KFRE values. Values with an asterisk* have been rounded up to the nearest 5 to maintain participant anonymity

		KFRE *n* (%)				
		< 5%	5–10%	10–15%	15–20%	> 20%
**Overall**		4,009 (82.9)	202 (4.2)	107 (2.2)	70 (1.5)	450 (9.3)
**Sex**	Female	2,343 (87.2)	93 (3.5)	43 (1.6)	24 (0.9)	184 (6.9)
	Male	1,666 (77.5)	109 (5.1)	64 (3.0)	46 (2.1)	266 (12.4)
**Age group**	18–40	29 (55.8)	6 (11.5)	5 (9.6)	5* (9.6)	7 (13.5)
	40–50	71 (49.3)	15 (10.4)	10 (6.9)	10* (6.9)	38 (26.4)
	50–60	212 (67.1)	20 (6.3)	8 (2.5)	5* (1.6)	71 (22.5)
	60–70	571 (73.8)	41 (5.3)	25 (3.2)	20* (2.6)	117 (15.1)
	70–80	1,623 (84.8)	66 (3.5)	41 (2.1)	30* (1.6)	153 (8.0)
	> 80	1,503 (91.2)	54 (3.3)	18 (1.1)	10* (0.6)	64 (3.9)
**SIMD group** Missing values = 137 (2.8%)	1–3 (most deprived)	1,770 (80.4)	105* (4.8)	50* (2.3)	37 (1.7)	239 (10.9)
	4–7	1,137 (84.6)	50* (3.7)	30* (2.2)	14 (1.0)	113 (8.4)
	8–10 (least deprived)	995 (84.9)	50* (4.3)	30* (2.6)	13 (1.1)	84 (7.2)
**Hypertension**	Yes	752 (95.4)	12 (1.5)	5 (0.6)	5* (0.6)	14 (1.8)
	No	3,257 (80.3)	190 (4.7)	102 (2.5)	70* (1.7)	436 (10.8)
**Diabetes**	Yes	1,367 (82.2)	54 (3.3)	37 (2.2)	37 (2.2)	168 (10.1)
	No	2,642 (83.2)	148 (4.7)	70 (2.2)	33 (1.0)	282 (8.9)

There were 594 people with incident kidney failure. Event rates per 1000 patient-years were 15.6 for eGFR < 15, 3.7 for kidney transplantation, 3.4 for long-term dialysis and 110.7 for all-cause mortality.

**Fig. 1:**
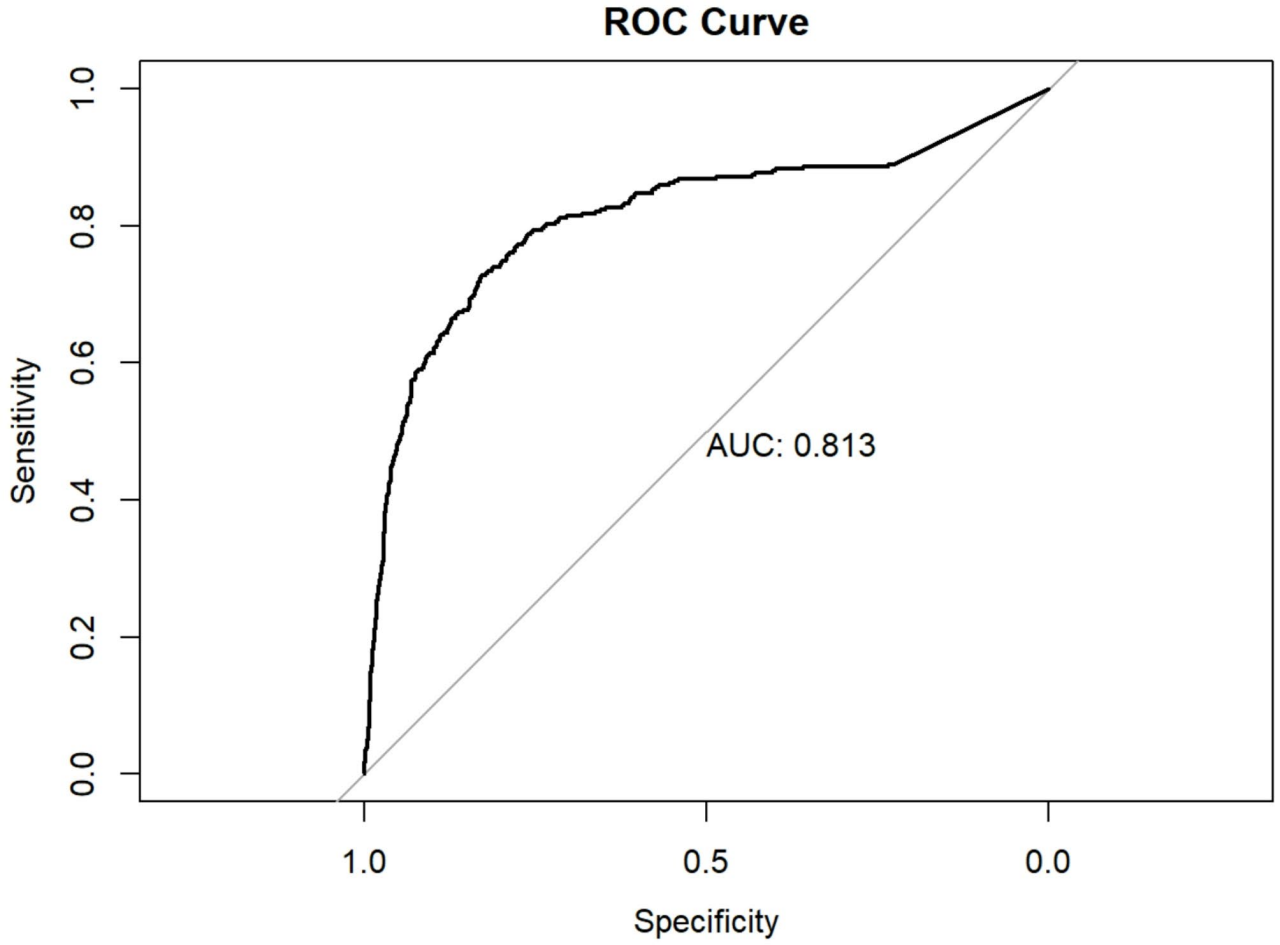
Area under receiver operating characteristic (ROC) curve (AUC) demonstrating KFRE’s discrimination at five years

**Fig. 2:**
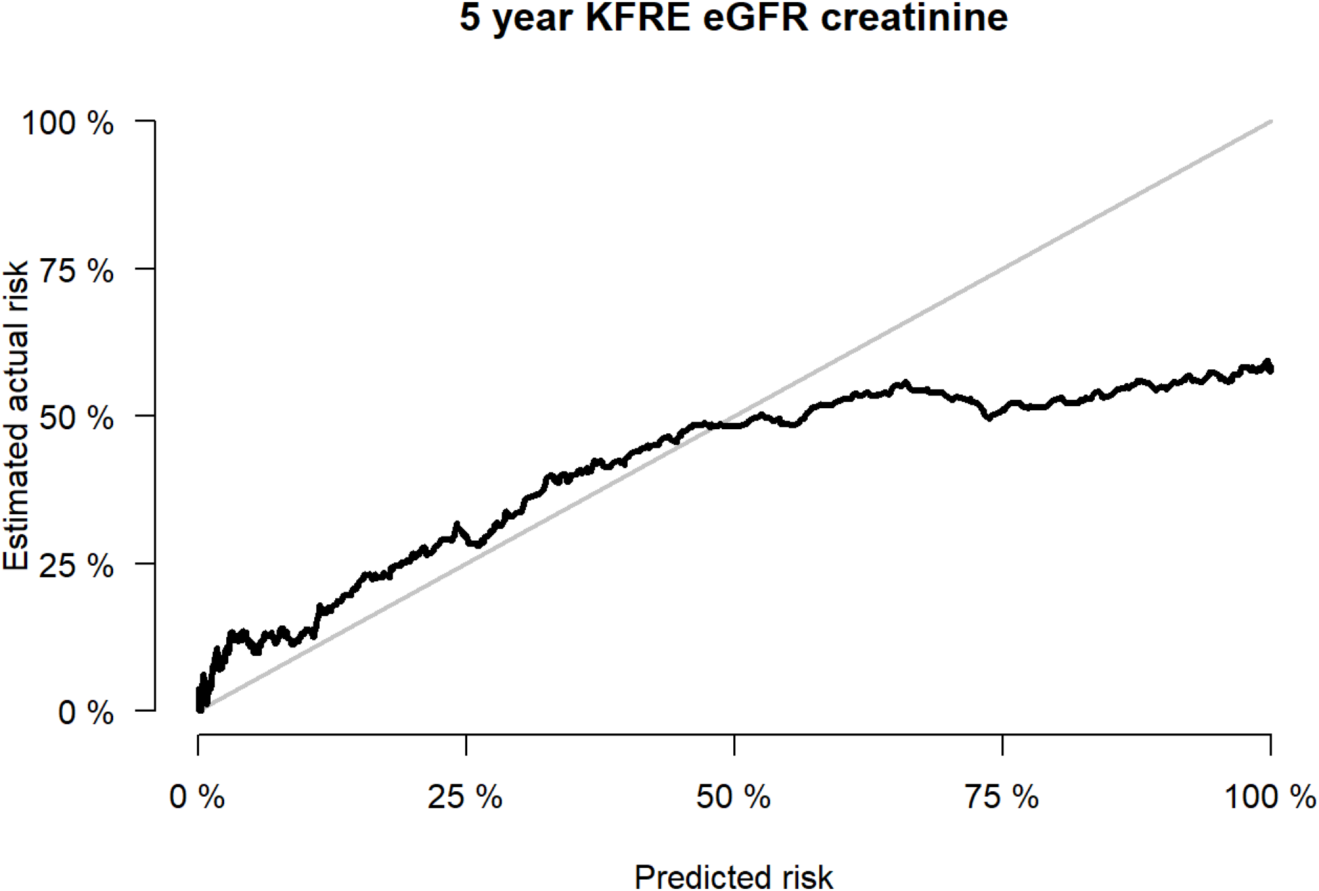
Calibration curve demonstrating KFRE’s calibration at five years

KFRE produced good discrimination at predicting kidney failure at five years with an area under the curve of 0.81 (Fig. 1). Assessment of KFRE’s calibration was also good, but with over-prediction of risk amongst higher risk groups (Fig. 2).

## Discussion

This study highlights that coverage of albuminuria testing is poor, with only 44.5% of the CKD population tested. This renders it impossible to calculate KFRE for most individuals in whom prognostication can help guide CKD management. Additionally, health inequalities have been highlighted with some groups being less likely to have uACR testing including females, younger adults, those without diabetes and those living in the most socioeconomically deprived areas.

The main strength of our study lies in its use of real-world, regional cohort perspective, with good representation across socioeconomic deprivation indices, allowing it to be generalisable to similar populations. In addition, a robust definition of kidney failure was utilised, including linked renal data. There are, however, several limitations. First, we lacked certain data, such as ethnicity, which is key when considering inequities and kidney health outcomes. Second, individuals with CKD were identified during the study period and it is not possible to say with certainty that they did not have CKD prior to this time point as we did not use a look-back period. Third, we restricted uACR testing to six months before or after CKD diagnosis; in some situations, uACR may be carried out at the first annual review 12 months after CKD diagnosis, although we feel this is suboptimal in assessing risk in a timely manner. Fourth, we did not know which patients were referred to secondary care renal services and when, to consider how clinical practice compared to guidelines. Finally, we were unable to assess the potential harms of uACR testing and the use of KFRE for referrals, such as extra GP workload, patient distress or excess treatment burden.

Previous studies have demonstrated lower rates of uACR testing than in this study: 37% of people with CKD living in England [18] and 41% of people with CKD in a US cohort [6]. CKD monitoring and risk stratification rely on uACR testing, so these low rates are concerning. There are a number of reasons which might explain these low rates: patients who are asked to submit urine samples for testing do not always do so, clinicians sometimes think that testing will not alter management [19], financial incentives for GPs testing people with diabetes in the UK were removed in 2014 [20], and primary care workloads may hinder chronic disease monitoring [21]. A possible avenue for increasing uACR testing is through mass screening [22]. A home-based uACR screening method trialled in the Netherlands achieved a favourable participation rate (51.8%), similar to other mass screening methods [23]. Although participation was lowest in those living in lower socioeconomic status areas [23]. It is likely that a multifaceted approach will be required to increase uACR testing rates, such as community engagement, support and education for patients and healthcare professionals.

In our study, people living in the most socioeconomically deprived areas had the highest uACR values and the highest rates of KRT (consistent with previous studies [24–26]), but they were the least likely to have uACR testing. Some studies have demonstrated an association between living in more deprived areas and the requirement for KRT, independent of other risk factors [25]. However, other studies suggest that this relationship may only exist in some ethnic groups [27], or not at all [28]. There is, however, good evidence that lower socioeconomic status is associated with presenting to kidney services late [29]. uACR testing rates in our study were low across all deprivation strata, but people living in deprived areas potentially have more to gain from improved CKD monitoring and management; a clear example of the Inverse Care Law [30]. Improving uACR testing rates and using KFRE to direct referrals to kidney services may help to reduce this inequity. Addressing these gaps in care may contribute to more of these people being prescribed guideline-directed therapies such as RAS-inhibitors and SGLT2-inhibitors, which have both reno- and cardio-protective effects [31].

We found that females, younger and older individuals, and those without diabetes were least likely to have uACR testing. It is well-recognised that people with diabetes are the most likely to be tested [5, 7, 32] as diabetes guidelines make clear recommendations for annual uACR testing [33]. In comparison, current hypertension guidelines are inconsistent with recommendations ranging from dipstick urine testing only to annual uACR testing [7]. Data from Sweden also show that women with CKD are less likely to have uACR testing than men [34]. It is unclear why these sex differences exist, but action should be taken to promote equitable access to CKD monitoring and management.

This study also highlights that incorporating KFRE into referral guidelines can help identify individuals at risk of kidney failure at an earlier time point than referrals based primarily on eGFR. This is in keeping other UK [16] and Canadian studies [35]. Earlier review in secondary care kidney clinics may allow for enhanced care planning for those who are likely to progress to kidney failure. However, the impact of KFRE on patient outcomes (such as reducing rates of kidney failure) does not yet appear to have been studied.

## Conclusions

KFRE effectively predicts the risk of kidney failure in individuals with CKD and can be used to guide referrals from primary care to renal clinics, but uACR testing rates must be improved to allow GPs to implement it effectively. Guideline-directed CKD monitoring and management is key for all populations, but there is evidence of particularly poor monitoring amongst some groups: females, younger adults and those without diabetes, with higher testing amongst those from the least socioeconomically deprived areas. Further research is required to elicit what barriers/factors are impacting poor uACR testing from both healthcare professional and patient perspectives. Additionally, there is an urgent need to develop interventions and appropriately targeted screening programmes based on these research gaps to improve uACR testing for individuals with, or at risk of, CKD.

## Electronic supplementary material

Below is the link to the electronic supplementary material.

Supplementary Material 1

## Data Availability

The data that support the findings of this study are not openly available due to reasons of sensitivity but access can be applied for through the West of Scotland Safe Haven (https://www.nhsggc.scot/hospitals-services/services-a-to-z/west-of-scotland-safe-haven/).

## Abbreviations

CKD: Chronic kidney disease
eGFR: Estimated glomerular filtration rate
KRT: Kidney replacement therapy
KFRE: Kidney failure risk equation
NICE: National Institute for Health Care Excellence
uACR: urine albumin–to–creatinine ratio
SCI: Scottish Care Information
SERPR: Strathclyde Electronic Renal Patient Register
SMR01: Scottish Morbidity Record 01
STROBE: Strengthening the Reporting of Observational Studies in Epidemiology
SIMD: Scottish Index of Multiple Deprivation
SD: Standard deviation
IQR: Inter–quartile range
aOR: Adjusted odds ratio
CI: Confidence interval

## References

[1] Bikbov B, Purcell CA, Levey AS, Smith M, Abdoli A, Abebe M, et al. Global, regional, and National burden of chronic kidney disease, 1990–2017: a systematic analysis for the global burden of disease study 2017. Lancet. 2020;395(10225):709–33. 32061315 10.1016/S0140-6736(20)30045-3 PMC7049905

[2] Phillips LA, Donovan KL, Phillips AO. Renal quality outcomes framework and eGFR: impact on secondary care. QJM-Int J Med. 2009;102(6):415–23. 10.1093/qjmed/hcp030 19349287

[3] Royston P, Altman DG. External validation of a Cox prognostic model: principles and methods. BMC Med Res Methodol. 2013;13:33. 23496923 10.1186/1471-2288-13-33 PMC3667097

[4] NICE. Chronic kidney disease: assessment and management 2021 guideline. 34672500

[5] Hull SA, Nitsch D, Caplin B, Griffith K, Wheeler DC. The National CKD audit: a primary care condition that deserves more attention. Br J Gen Practice: J Royal Coll Gen Practitioners. 2018;68(673):356. 10.3399/bjgp18X697997 PMC6058639 30049752

[6] Chu CD, Powe NR, Shlipak MG, Scherzer R, Tummalapalli SL, Estrella MM, et al. Albuminuria testing and nephrology care among insured US adults with chronic kidney disease: a missed opportunity. BMC Prim Care. 2022;23(1):299. 36434513 10.1186/s12875-022-01910-9 PMC9700954

[7] Shin J-I, Chang AR, Grams ME, Coresh J, Ballew SH, Surapaneni A, et al. Albuminuria testing in hypertension and diabetes: an Individual-Participant data Meta-Analysis in a global consortium. Hypertension. 2021;78(4):1042–52. 34365812 10.1161/HYPERTENSIONAHA.121.17323 PMC8429211

[8] Vanholder R, Annemans L, Braks M, Brown EA, Pais P, Purnell TS, et al. Inequities in kidney health and kidney care. Nat Rev Nephrol. 2023;19(11):694–708. 37580571 10.1038/s41581-023-00745-6

[9] West of Scotland Safe Haven. - NHSGGC [Available from: https://www.nhsggc.scot/hospitals-services/services-a-to-z/west-of-scotland-safe-haven/

[10] Hyslop A, Robertson K. Integrating clinical information in NHS Scotland: the role of Scottish care information store. Inf Prim Care. 2004;12(2):103–7. 15319063

[11] The Scottish Care Information - Diabetes Collaboration. (SCI-DC) [Available from: https://www.sci-diabetes.scot.nhs.uk/

[12] von Elm E, Altman DG, Egger M, Pocock SJ, Gøtzsche PC, Vandenbroucke JP. The strengthening the reporting of observational studies in epidemiology (STROBE) statement: guidelines for reporting observational studies. J Clin Epidemiol. 2008;61(4):344–9. 18313558 10.1016/j.jclinepi.2007.11.008

[13] Collins GS, Reitsma JB, Altman DG, Moons KG. Transparent reporting of a multivariable prediction model for individual prognosis or diagnosis (TRIPOD): the TRIPOD statement. J Clin Epidemiol. 2015;68(2):134–43. 25579640 10.1016/j.jclinepi.2014.11.010

[14] Scottish Government Scottish Index. of Multiple Deprivation [Available from: https://www2.gov.scot/Topics/Statistics/SIMD

[15] NICE. CKD 2015 guideline.

[16] Major RW, Shepherd D, Medcalf JF, Xu G, Gray LJ, Brunskill NJ. The kidney failure risk equation for prediction of end stage renal disease in UK primary care: an external validation and clinical impact projection cohort study. PLoS Med. 2019;16(11):e1002955. 31693662 10.1371/journal.pmed.1002955 PMC6834237

[17] Levey AS, Stevens LA, Schmid CH, Zhang YL, Castro AF 3rd, Feldman HI, et al. A new equation to estimate glomerular filtration rate. Ann Intern Med. 2009;150(9):604–12. 19414839 10.7326/0003-4819-150-9-200905050-00006 PMC2763564

[18] Fraser SD, Parkes J, Culliford D, Santer M, Roderick PJ. Timeliness in chronic kidney disease and albuminuria identification: a retrospective cohort study. BMC Fam Pract. 2015;16:18. 25879207 10.1186/s12875-015-0235-8 PMC4333177

[19] Abdel-Kader K, Greer RC, Boulware LE, Unruh ML. Primary care physicians’ familiarity, beliefs, and perceived barriers to practice guidelines in non-diabetic CKD: a survey study. BMC Nephrol. 2014;15:64. 24755164 10.1186/1471-2369-15-64 PMC4021215

[20] Minchin M, Roland M, Richardson J, Rowark S, Guthrie B. Quality of care in the united Kingdom after removal of financial incentives. N Engl J Med. 2018;379(10):948–57. 30184445 10.1056/NEJMsa1801495

[21] Fisher RFR, Croxson CHD, Ashdown HF, Hobbs FDR. GP views on strategies to Cope with increasing workload: a qualitative interview study. Br J Gen Practice: J Royal Coll Gen Practitioners. 2017;67(655):e148. 10.3399/bjgp17X688861 PMC5308121 28093421

[22] Lamprea-Montealegre JA, Estrella MM. Population-wide albuminuria screening: implications for CKD detection and management. Lancet. 2023;402(10407):1020–1. 37597525 10.1016/S0140-6736(23)01140-6

[23] van Mil D, Kieneker LM, Evers-Roeten B, Thelen MHM, de Vries H, Hemmelder MH, et al. Participation rate and yield of two home-based screening methods to detect increased albuminuria in the general population in the Netherlands (THOMAS): a prospective, randomised, open-label implementation study. Lancet. 2023;402(10407):1052–64. 37597522 10.1016/S0140-6736(23)00876-0

[24] Fraser SD, Roderick PJ, Aitken G, Roth M, Mindell JS, Moon G, et al. Chronic kidney disease, albuminuria and socioeconomic status in the health surveys for England 2009 and 2010. J Public Health (Oxf). 2014;36(4):577–86. 24277777 10.1093/pubmed/fdt117

[25] Hossain MP, Palmer D, Goyder E, El Nahas AM. Association of deprivation with worse outcomes in chronic kidney disease: findings from a hospital-based cohort in the united Kingdom. Nephron Clin Pract. 2012;120(2):c59–70. 22269817 10.1159/000334998

[26] Kar D, El-Wazir A, Delanerolle G, Forbes A, Sheppard JP, Nath M, et al. Predictors and determinants of albuminuria in people with prediabetes and diabetes based on smoking status: A cross-sectional study using the UK biobank data. EClinicalMedicine. 2022;51:101544. 35813092 10.1016/j.eclinm.2022.101544 PMC9256818

[27] Merkin SS, Coresh J, Diez Roux AV, Taylor HA, Powe NR. Area socioeconomic status and progressive CKD: the atherosclerosis risk in communities (ARIC) study. Am J Kidney Dis. 2005;46(2):203–13. 16112038 10.1053/j.ajkd.2005.04.033

[28] Solbu MD, Thomson PC, Macpherson S, Findlay MD, Stevens KK, Patel RK, et al. Serum phosphate and social deprivation independently predict all-cause mortality in chronic kidney disease. BMC Nephrol. 2015;16:194. 26627078 10.1186/s12882-015-0187-1 PMC4666082

[29] Bello AK, Peters J, Rigby J, Rahman AA, El Nahas M. Socioeconomic status and chronic kidney disease at presentation to a renal service in the united Kingdom. Clin J Am Soc Nephrol. 2008;3(5):1316–23. 18579673 10.2215/CJN.00680208 PMC2518794

[30] Watt G. The inverse care law revisited: a continuing blot on the record of the National health service. Br J Gen Practice: J Royal Coll Gen Practitioners. 2018;68(677):562–3. 10.3399/bjgp18X699893 PMC6255247 30498141

[31] Neuen BL, Ohkuma T, Neal B, Matthews DR, de Zeeuw D, Mahaffey KW, et al. Relative and absolute risk reductions in cardiovascular and kidney outcomes with Canagliflozin across KDIGO risk categories: findings from the CANVAS program. Am J Kidney Dis. 2021;77(1):23–e341. 32971190 10.1053/j.ajkd.2020.06.018

[32] Sullivan MK, Jani BD, Rutherford E, Welsh P, McConnachie A, Major R et al. Potential impact of NICE guidelines on referrals from primary care to nephrology. Br J Gen Pract. 2022:BJGP.2022.0145. 10.3399/BJGP.2022.0145 PMC9678375 36376072

[33] Cosentino F, Grant PJ, Aboyans V, Bailey CJ, Ceriello A, Delgado V, et al. 2019 ESC guidelines on diabetes, pre-diabetes, and cardiovascular diseases developed in collaboration with the EASD. Eur Heart J. 2020;41(2):255–323. 31497854 10.1093/eurheartj/ehz486

[34] Swartling O, Yang Y, Clase CM, Fu EL, Hecking M, Hödlmoser S, et al. Sex differences in the recognition, monitoring, and management of CKD in health care: an observational cohort study. J Am Soc Nephrol. 2022;33(10):1903–14. 35906075 10.1681/ASN.2022030373 PMC9528319

[35] Hingwala J, Wojciechowski P, Hiebert B, Bueti J, Rigatto C, Komenda P, et al. Risk-Based triage for nephrology referrals using the kidney failure risk equation. Can J Kidney Health Dis. 2017;4:2054358117722782. 28835850 10.1177/2054358117722782 PMC5555495

