# Supplementary material for: Analysis of the kidney failure risk equation implementation in routine clinical practice and health inequalities in chronic kidney disease care: a retrospective cohort study
Source: BMC Nephrol. 2025 Mar 4;26:113. doi: 10.1186/s12882-025-04043-0 (PMC11881359; doi:10.1186/s12882-025-04043-0)
Supplement: Supplementary file 1 — Supplementary Material 1 [file 12882_2025_4043_MOESM1_ESM.docx]

Additional File 1.

Figure S1. Analysis 1 CONSORT patient flow diagram showing patient inclusion and exclusions


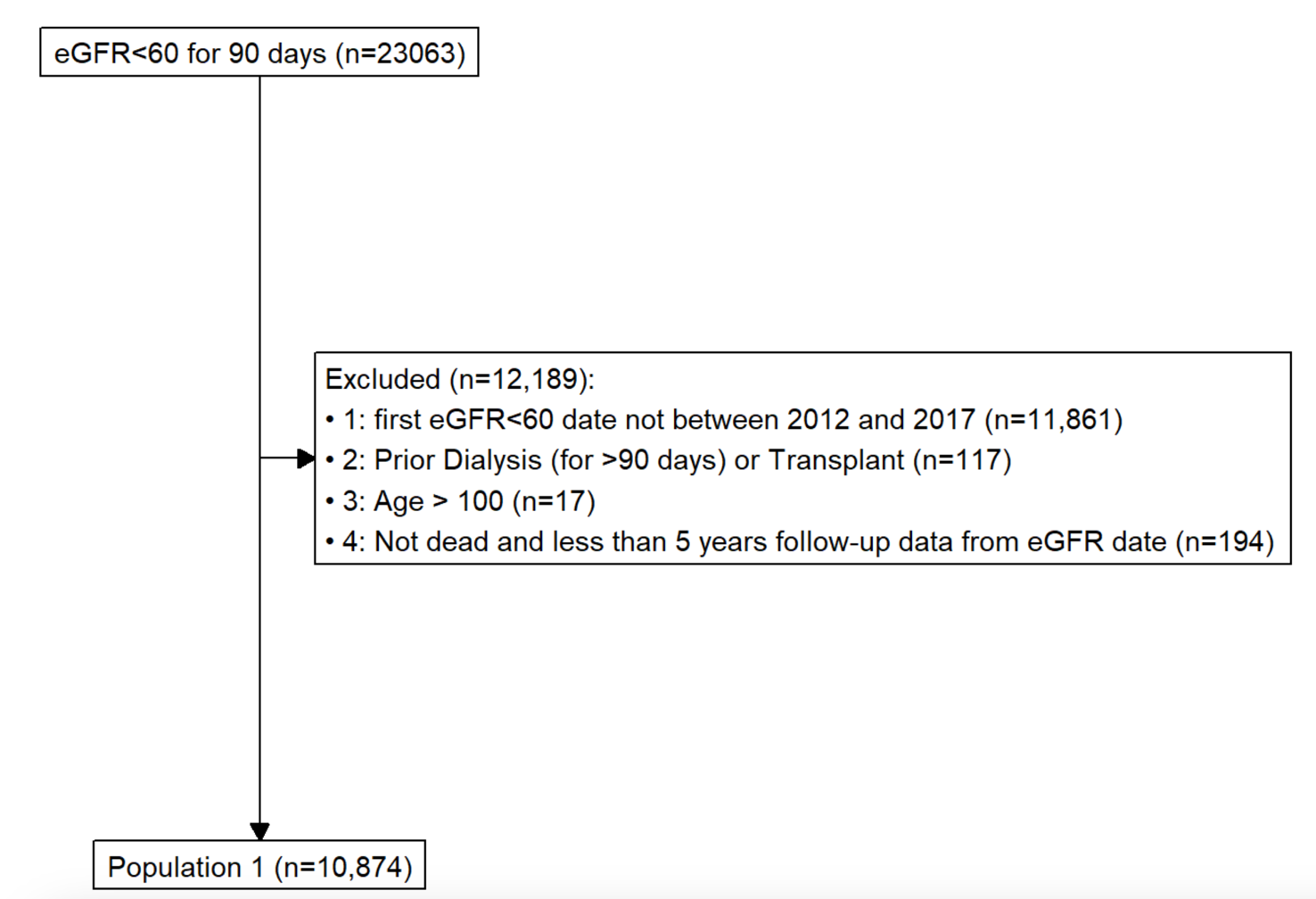


Figure S2. Analysis 2 CONSORT patient flow diagram showing patient inclusion and exclusion


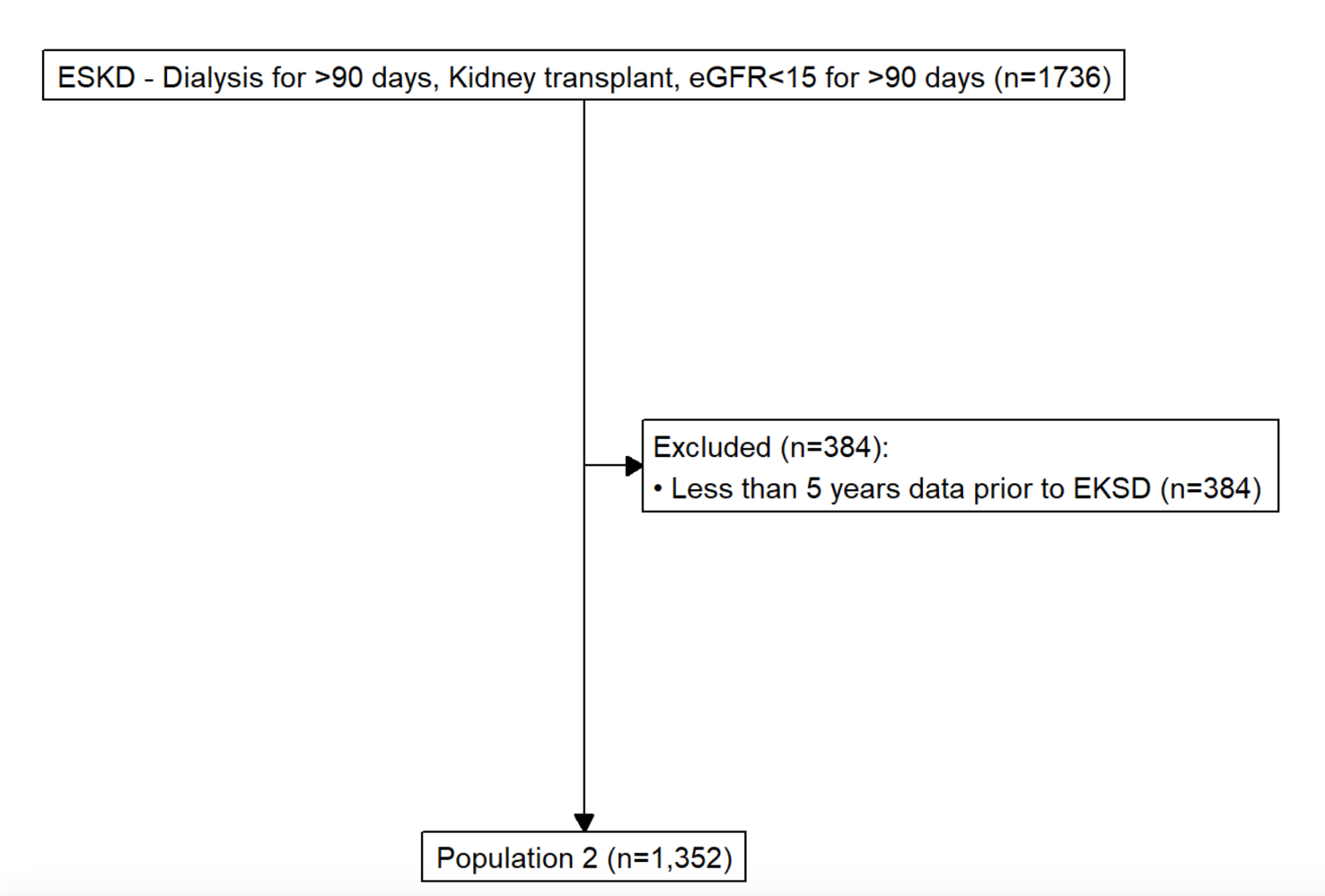


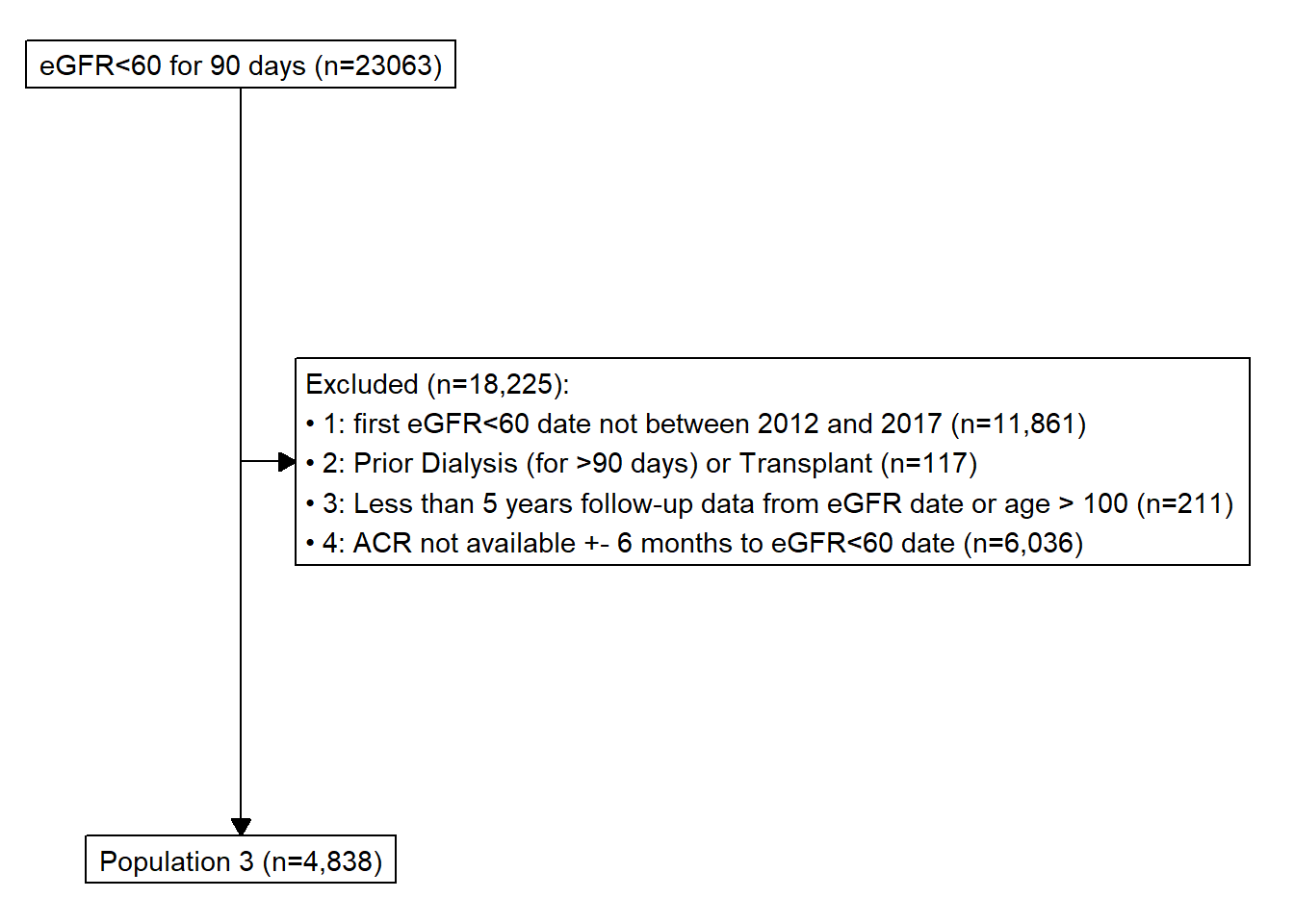
Figure S3. Analysis 3 CONSORT patient flow diagram showing patient inclusion and exclusions
